# Reading Character from Inspection of the Vulva and Vagina

**Published:** 1899-08

**Authors:** 


### *EDITORIAL NOTES AND COMMENTS.*

The Business office of THE JOURNAL-RECORD is 305, 306 Fitten Building.

The Editorial office is 318-319-320 The Prudential.

Address all Business communications to Dr. M. B. Hutchins, Mgr.

Make remittances payable to THE ATLANTA JOURNAL-RECORD OF MEDICINE.

On matters pertaining to the Editorial and Original Communications address Dr. Bernard Wolff, Atlanta.

Reprints of original articles will be furnished at cost price. Requests for the same should always be made *on the manuscript.*

We will present, post-paid, on request, to each contributor of an original article, twenty (20) marked copies of THE JOURNAL-RECORD containing such article.

### READING CHARACTER FROM INSPECTION OF THE VULVA AND VAGINA.

PHRENOLOGY and palmistry are mild delusions which have acquired a certain amount of respectability through age. They are popular, not because any one actually believes them true, but because they concern one's self, an ever-fascinating, fresh and blooming subject.

The head and the hands have hitherto enjoyed a monopoly as indices of character, but now a surgeon of the "orificial" cult has added the female external organs of generation to the list.

The patient, before undergoing the various operations necessitated by her neurasthenic condition, is subjected to a "tissue-reading."

The following is an example, taken from the *Journal of Orificial Surgery:* Miss E., age 24. "Pelvic conditions: Adherent prepuce; hypertrophy labia minora; hymen mounted by small papillæ; retroversion; vagina rough; fine papillæ and fine pockets in rectum."

The "genitologist" strikes an attitude and throws this over his shoulder to his attentive audience: "Upon inspection, the large size of the labia minora and the hood of the clitoris, together with the healthful color of the parts, indicate superb nutrition, point to unlimited energy. This must be a woman of great power. She can accomplish anything she undertakes, and whatever she does is done with great determination and energy. At the same time she is as sensitive as a child. Nothing escapes her observation, and she is easily pleased or injured. The morbid sensitiveness and fineness of her nature are indicated by the toothlike projections along the margin of the hymen, which are exceedingly fine and sensitive. So while she has the power of an organizer and campaigner, a successful schemer, she at the same time possesses wonderful powers of observation, and nothing escapes her notice. It is rare to find these opposite qualities in a single individual, and when they occur they mark a phenomenal nature. These people are either away up or away down. They belong to the inspirational type of human nature, and frequently see visions. This woman could be a Jeanne d' Arc. At the same time she could be a seamstress, and if she had choice of work it would be of the finer type of sewing. She could head a seminary of learning (as a matter of fact, she functionated—this woman of such infinite variety—as teacher in the primary grade of a public school), or could give lessons in a most successful manner to a single pupil. Her nature is more or less tempest-tossed, as is evidenced by the physical conditions under observation, and she is at present wasting much of her power, in one direction in too ambitious dreams, and in the other in fretful particulars of every-day life. By orificial work we can remove her temptations and restore her to a proper balance of mind and body, and when she finds proper scope for her powers she should achieve greatness in any direction in which her ambitions direct her."

These observations were amply verified by those who knew her, and who freely averred that she could "stoop to pick up a flower and could also lead cavalry." All this because of a pouting vulva and a spiny hymen, a condition which, to the uninitiated, would suggest masturbation, but to the observation of the orificial surgeon indicates extreme force of character. If a sensitive hymen indicates superior mentality, by the same reasoning an urethral caruncle suggests genius too lofty for the finite mind to grasp.

"We have before us material for volumes of thought, out of which should come light to every physician that reads."

Not only that, but what is more to the point, the surgeon, while swabbing out the vagina, might be able to glean from a study of the vulva some notion of the patient's ability to pay.

